# Widely Targeted Metabolomics Analysis Revealed the Component Differences of *Hemerocallis citrina* Borani in Different Production Areas of Datong

**DOI:** 10.3390/foods13213404

**Published:** 2024-10-25

**Authors:** Yongxia Fu, Haizhen Wang, Zhenyu Liu, Han Wang, Mengying Zhao, Zhihao Li, Shang Guo

**Affiliations:** 1 Shanxi Institute for Functional Food, Shanxi Agricultural University, Taiyuan 030031, China; wanghaizhen0505@163.com (H.W.); zhaomy1128@163.com (M.Z.); 18834716156@163.com (Z.L.); 2 College of Horticulture, Shanxi Agricultural University, Jinzhong 030801, China; 3 College of Biomass Science & Engineering, Sichuan University, Chengdu 610065, China; sculzy2018@163.com; 4 College of Food Science and Nutritional Engineering, China Agricultural University, Beijing 100083, China; wh18396506285@163.com; 5 College of Veterinary Medicine, Hunan Agricultural University, Changsha 410128, China

**Keywords:** *Hemerocallis citrina* Borani, widely targeted metabolomics, differential metabolites, metabolic pathway

## Abstract

*Hemerocallis citrina* Borani (*H. citrina*) has garnered significant attention due to its abundant nutritional quality. Datong, located in Shanxi Province, is recognized as one of the four major production regions for high-quality *H. citrina*. While Datong boasts multiple production areas, the nutritional composition of daylilies varies across regions due to environmental factors and planting patterns, which remain unclear. This study focuses on the total polyphenol and flavonoid contents (TPCs and TFCs) and protein content of *H. citrina* from three areas in Datong: Sanshilipu (DTSSLP), Dangliuzhuang (DTDLZ), and Jijiazhuang (DTJJZ). Additionally, a widely targeted metabolomics analysis was used to analyze the metabolite composition of *H. citrina* from these three areas. The results showed that *H. citrina* in DTSSLP had the highest contents of protein and amino acids, as well as TPCs and TFCs. A total of 798 differential metabolites were identified in *H. citrina* across the areas, with DTSSLP showing the highest levels of different classifications of metabolites, indicating its enhanced health benefits and physiological activities. Nine metabolic pathways were related with the different characteristics among DTSSLP, DTDLZ, and DTJJZ. This study provides theoretical support for distinguish *H. citrina* from different producing regions and elucidates the mechanisms underlying its metabolic pathways.

## 1. Introduction

*Hemerocallis citrina* Borani (*H. citrina*) is a perennial herb belonging to the Liliaceae family, originally native to central and northern China, the Korean Peninsula, and Japan [1,2]. Renowned for its vibrant color, substantial flesh, and delightful taste, *H. citrina* holds a distinguished position as one of the “Four Treasures of Vegetables” [3,4,5]. The edible part of *H. citrina*, namely its flower buds, is abundantly endowed with various nutrients and bioactive compounds. These include polysaccharides, amino acids, proteins, and flavonoids, making it a valuable nutritional resource. Studies have indicated its potential to improve sleep quality [6]. Additionally, both water and ethanol extracts of *H. citrina* have demonstrated antidepressant properties, with rutin in the water extract being one of the main antidepressant components [7]. Moreover, freeze-dried *H. citrina* has been found to improve insufficient lactation in rats, with flavonoids and phenolic acids potentially being the active components that promote lactation [8]. Thus, *H. citrina* is receiving increasing attention for its rich nutritional benefits.

Datong, located in Shanxi province, is recognized as one of the four major production regions for *H. citrina*. The volcanic soil in this region is exceptionally rich in essential nutrients crucial for optimal plant growth. Moreover, the substantial temperature fluctuations between day and night, coupled with ample rainfall in Datong, provide highly favorable conditions for cultivating *H. citrina*, thereby enhancing its overall quality and nutritional value [9]. Several standardized production bases have been established, including those in Sanshilipu Village (DTSSLP), Jijiazhuang Village (DTJJZ), and Dangliuzhuang Village (DTDLZ). While *H. citrina* from different production areas may appear similar in appearance, their nutritional profiles can vary significantly due to factors such as altitude and temperature, and cultivation techniques. With the increasing market demand for *H. citrina*, it becomes crucial to analyze the nutritional quality characteristics of *H. citrina* sourced from different regions. This analysis can enable consumers to effectively discern the differences between *H. citrina* originating from various geographical sources.

Metabolomics is the science of studying the types, quantities, and changes of endogenous metabolites. Metabolomics techniques can reveal the specificity of different varieties, the formation of vegetable quality, and biological response mechanisms [10]. With the continuous development of metabolomics detection technology, an increasing number of metabolites have been identified. Techniques such as ultra-performance liquid chromatography–tandem mass spectrometry (UPLC–MS/MS) and gas chromatography–tandem mass spectrometry (GC–MS/MS) are widely used in the identification of vegetable quality components and the analysis of key post-harvest metabolites. Also, nuclear magnetic resonance (NMR) has also been reported as one of the tools for metabolomics analysis [11,12,13], which can be used to study changes in vegetable quality [14,15]. This study selected *H. citrina* from DTSSLP, DTJJZ, and DTDLZ in Datong and conducted a widely targeted metabolomics using UPLC–MS/MS to evaluate the quality characteristics of *H. citrina* from these three production areas.

## 2. Materials and Methods

### 2.1. H. citrina Materials

Fresh *H. citrina* were harvested in July 2022 from DTSSLP (113.447° E, 40.139° N), DTDLZ (113.494° E, 39.872° N), and DTJJZ (113.456° E, 39.850° N) in Datong City, Shanxi Province, China. The climatic conditions data of the considered areas in the year before harvesting are presented in Supplementary Table S1. The *H. citrina* flowers were picked between 6 and 7 a.m., and mature *H. citrina* buds were selected based on their color, shape, and size. After harvesting, they were immediately flash-frozen with liquid nitrogen and then transported in dry ice to the laboratory for storage. The harvested *H. citrina* samples were vacuum freeze-dried, pulverized into powder, and stored at a desiccator for future use.

### 2.2. Chemical Composition Analyses

Protein content was determined using the Kjeldahl method with a conversion factor of 6.25 for nitrogen-to-protein conversion [16]. Total polyphenol content (TPC) and total flavonoid content (TFC) were determined according to the methods proposed previously [17,18].

To analyze amino acid contents, 0.1 g (accurate to 0.0001 g) of dried *H. citrina* powder was weighed and placed into a hydrolysis tube. Subsequently, 10 mL of 6 mol/L hydrochloric acid solution and 50 μL of phenol solution were added. The hydrolysis tube was then cooled in an ice bath for 3–5 min under nitrogen flushing. Then, it was sealed and placed in a 110 °C electric heating air-blast drying oven for 22 h for hydrolysis. After cooling to room temperature, the hydrolysate was filtered using a 0.45 μm filter membrane and transferred into a 50 mL volumetric flask. The hydrolysis tube was rinsed with distilled water several times to ensure complete transfer, and then made up to volume. An amount of 1 mL of the filtrate was transferred into a test tube with precision, followed by evaporation to dryness at 45 °C using a parallel vacuum evaporator. Subsequently, the residual material was further dried with an additional 1 mL of water. The desiccated residue in the test tube was then dissolved in pH 2.2 citric acid buffer solution through agitation, and the resultant solution was filtered through a 0.22 μm filter membrane for subsequent analysis. Amino acid (AA) content was quantified using an automated amino acid analyzer equipped with a chromatography column containing sulfonic acid-type cation exchange resin (Sykam Technologies, Munich, Bavaria, Germany), with detection wavelengths set at 570 nm and 440 nm. Total amino acid (TAA) and essential amino acid (EAA) contents were subsequently calculated.

### 2.3. Pre-Treatment of H. citrina Samples

The *H. citrina* samples underwent pulverization into a powder at 30 Hz for 1.5 min. Following this, 50 mg of the powdered sample was blended with 1200 μL of a 70% methanol–water internal standard extraction solution that had been pre-cooled to −20 °C. The mixture was then agitated by vortexing for 30 s every 30 min, with this cycle repeated six times. Subsequently, the mixture was centrifuged at 12,000 rpm for 3 min. The resulting supernatant was collected, passed through a 0.22 μm micropore filter, and the filtered sample was preserved in a vial for subsequent analysis using UPLC and MS/MS(Sciex Applied Biosystems, Frisco, TX, USA).

### 2.4. UPLC Analysis

The UPLC conditions were set as follows: An Agilent chromatographic column SB-C18 (1.8 μm, 2.1 mm × 100 mm) was used, with mobile phase A composed of ultrapure water containing 0.1% formic acid, and mobile phase B consisting of acetonitrile containing 0.1% formic acid. The elution gradient began with 5% of phase B, linearly increasing to 95% over 9.00 min, and maintained at 95% for 1 min. From 10.00 to 11.10 min, phase B was reduced to 5% and maintained for 14 min. The flow rate was set at 0.35 mL/min, with a column temperature of 40 °C and an injection volume of 2 μL.

The mass spectrometry conditions included an electrospray ionization (ESI) source with temperature set at 500 °C. In positive ion mode, the ion spray voltage (IS) was 5500 V, and in negative ion mode, it was −4500 V. Gas settings for ion source gas I (GS), gas II (GSII), and curtain gas (CUR) were 50, 60, and 25 psi, respectively. Collision-induced ionization parameters were set to high, and QqQ scans were conducted using multiple reaction monitoring (MRM) mode with nitrogen gas as the collision gas set to medium. In the MRM mode, the first quadrupole (Q1) selected the precursor ions (parent ions) of the target substance based on the set mass-to-charge ratio range and filtered out ions corresponding to other molecular weights to initially eliminate interference. The second quadrupole (Q2), also known as the collision cell, induces ionization of the precursor ions in the collision chamber, causing them to fragment into many product ions. These product ions were then filtered by the triple quadrupole to select one characteristic product ion, with the third quadrupole (Q3) used to analyze the product ions generated in the collision cell. By eliminating interference from non-target ions, the quantification becomes more accurate, and reproducibility is improved. Declustering potential (DP) and collision energy (CE) for individual MRM transitions were optimized further. A specific set of MRM transitions (pairs of Q1 and Q3) was monitored during each period according to the metabolites eluted within that period.

### 2.5. Qualitative and Quantitative Analysis of Metabolites

After obtaining metabolomics data from different samples, qualitative and quantitative mass spectrometry analysis of the metabolites in the samples was performed based on the Metware Database (MWDB). First, the substance information was matched in the MWDB3.0 database using the information detected by MRM (secondary mass spectrometry including all fragment ions of the substance, retention time (RT), Q1, Q3, DP, and CE). During the analysis, isotopic signals, duplicate signals containing K^+^, Na^+^, and NH_4_^+^ ions, as well as duplicate signals from fragment ions of larger molecules, were removed. The qualitative analysis was divided into three levels: the first level required a matching score of 0.7 or higher between the secondary mass spectrum, RT, and the database of the sample; the second level required the sample material’s secondary mass spectrometry, with an RT and database matching score of 0.5–0.7; and the third level indicated that the sample material’s Q1, Q3, RT, DP, and CE are consistent with those in the database.

The quantification of metabolites was based on the RT of different metabolites, characteristic ions for each substance were screened using triple quadrupole filtering, and the signal intensity (count per second, CPS) of the characteristic ions was obtained in the detector. Then, mass spectrometry files of samples were opened in MultiQuant^TM^ 3.0 software, where the RT and CPS data were used for chromatographic peak integration and correction, yielding the peak area of each chromatographic peak, which represents the relative content of a specific substance in the sample. Finally, all chromatographic peak area integration data were exported and saved [19].

### 2.6. Statistical Analysis

All experiments were conducted in triplicate. One-way analysis of variance (ANOVA) was performed using SPSS 22 (SPSS Inc., Chicago, IL, USA), with Duncan’s multiple range test applied at a 5% confidence level. Statistical analysis of metabolite data from *H. citrina* samples included hierarchical cluster analysis (HCA), principal component analysis (PCA), and orthogonal partial least-squares discrimination analysis (OPLS-DA). HCA visually depicted the distribution of metabolites and samples, while PCA illustrated metabolic variances among different sample groups. The OPLS-DA model enabled comparison of metabolite compositions across various production regions of *H. citrina*. Differential metabolites were selected based on variable importance in projection (VIP) values > 1 and log2 (fold change, FC) > 2. Subsequently, metabolites were annotated based on the KEGG compound database (https://www.kegg.jp/kegg/compound/ (19 September 2024)) and mapped to KEGG pathway databases (https://www.kegg.jp/kegg/pathway.html (19 September 2024)), which were accessed on December 1995) [20]. Pathway enrichment analysis was further conducted on significantly altered metabolites using hypergeometric testing to identify trends.

## 3. Results and Discussion

### 3.1. Analysis of Nutritional Composition of H. citrina from Different Production Areas of Datong

Amino acids, flavonoids, and phenolic acids are abundant in medicinal and food-related plants, playing crucial roles in our daily dietary intake. The TFC, TPC, and AAs of *H. citrina* from different production areas of Datong are shown in Table 1 and Table 2. The TPCs, TFCs, and protein contents in DTSSLP were significantly higher than that in DTDLZ and DTJJZ (*p* < 0.05). In addition, DTSSLP had the highest TAAs and EAAs, followed by DTDLZ and DTJJZ, which corresponded to their protein contents.

### 3.2. Multivariate Statistical Analysis

**Figure 1:**
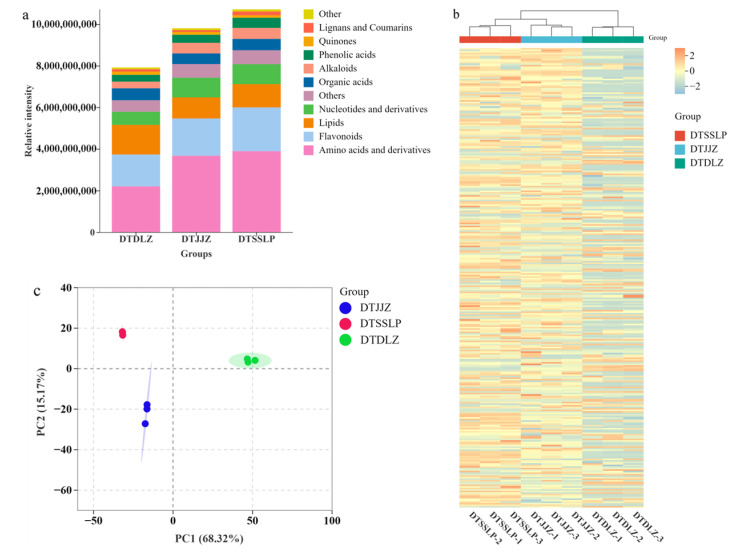
(**a**) Relative proportions of different categories of metabolites in *H. citrina* from different production areas; (**b**) PCA score plot; (**c**) hierarchical cluster analysis.

Firstly, we conducted an analysis and comparison of the metabolite composition proportions in *H. citrina* sourced from different production areas, which is illustrated in Figure 1a. Amino acids and derivatives, flavonoids, and lipids emerged as the predominant metabolite categories in *H. citrina* from these regions. Among these, *H. citrina* from DTSSLP exhibited the highest levels of amino acids and derivatives, as well as flavonoids, correlating with its elevated amino acid and total flavonoid content. Amino acids and derivatives are essential to plant growth and response to environmental challenges [21]. Moreover, amino acids can be precursors for numerous primary and secondary metabolites, essential for human health and pivotal in shaping the taste profile of *H. citrina* [22,23]. Many flavonoids, such as quercetin, also benefit human health [24]. Thus, *H. citrina* of DTSSLP may have better flavor, health benefits, and the ability to resist environmental stress. Moreover, lipid compounds play several important physiological roles in daylily growth and development, such as energy storage and acting as signaling molecules [25]. In this study, *H. citrina* from DTDLZ had the highest lipid content, indicating that it may have anti-inflammatory effects. Furthermore, HCA was used to analyze the differences and similarities in metabolite profiles among *H. citrina* from the three areas, with each group being biologically replicated three times to demonstrate the robustness and reliability of the data. The findings showed that the metabolite profiles of *H. citrina* from DTSSLP and DTJJZ demonstrated a higher degree of similarity, followed by DTDLZ (Figure 1b). Similarly, PCA was utilized to explore the overall distinctions among metabolite categories and the variability within each sample group (Figure 1c). Principal components PC1 and PC2 accounted for 68.32% and 15.17% of the variance in the original dataset, respectively. The distinct separation of *H. citrina* samples from the three areas indicated pronounced differences in the metabolite compositions among *H. citrina* from various production regions.

### 3.3. OPLS-DA of Differential Metabolic Analyses

**Figure 2:**
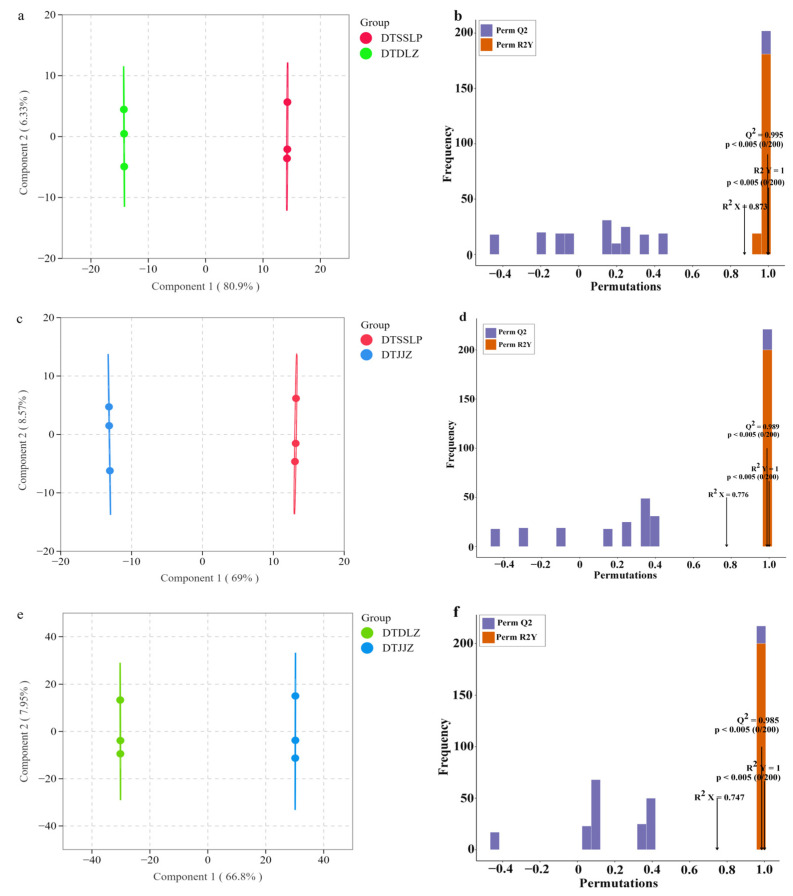
(**a**,**c**,**e**) OPLS-DA score chart of DTDLZ vs. DTSSLP, DTJJZ vs. DTSSLP, and DTDLZ vs. DTJJZ comparison groups; (**b**,**d**,**f**) OPLSDA model permutation test for DTSSLP vs. DTDLZ, DTSSLP vs. DTJJZ, and DTDLZ vs. DTJJZ.

The OPLS-DA analysis was conducted to investigate the differential metabolites in *H. citrina* from three production regions. As depicted in Figure 2, three parameters—R2X, R2Y, and Q2—were employed to assess the OPLS-DA model. A Q2 value exceeding 0.5 signifies an effective model, with a Q2 value surpassing 0.9 indicating a high level of model stability [26]. In this study, the R2X and R2Y for DTSSLP and DTJJZ were 0.776 and 1 (Figure 2d), respectively; for DTSSLP and DTDLZ, they were 0.873 and 1 (Figure 2b); and for DTJJZ and DTDLZ, they were 0.747 and 1 (Figure 2f). The Q2 values for all three comparison groups exceeded 0.9, confirming the robust stability of the OPLS-DA model. In addition, the OPLS-DA score plot showed that *H. citrina* from DTJJZ, DTDLZ, and DTSSLP were distinctly separated from each other (Figure 2a,c,e), suggesting significant differences in metabolite composition among *H. citrina* from different producing areas.

### 3.4. Differential Metabolite Profiling of Three Comparison Groups

**Figure 3:**
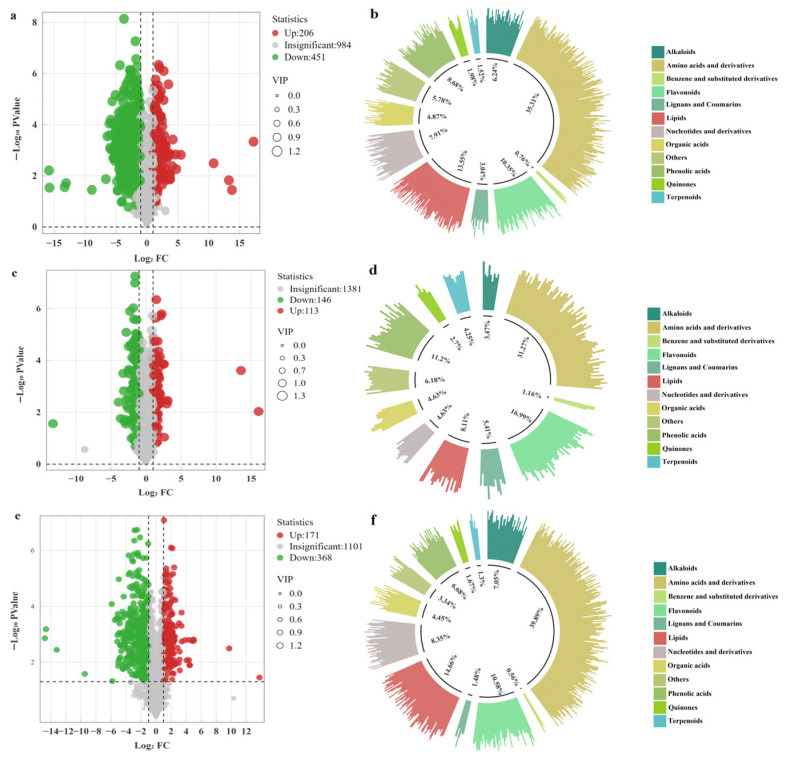
(**a**,**c**,**e**) Volcano plots of the quantity of differential metabolites in the comparison groups of DTDLZ vs. DTSSLP, DTJJZ vs. DTSSLP, as well as DTDLZ vs. DTJJZ. (**b**,**d**,**f**) Classification of differential metabolites in different comparison groups.

**Figure 4:**
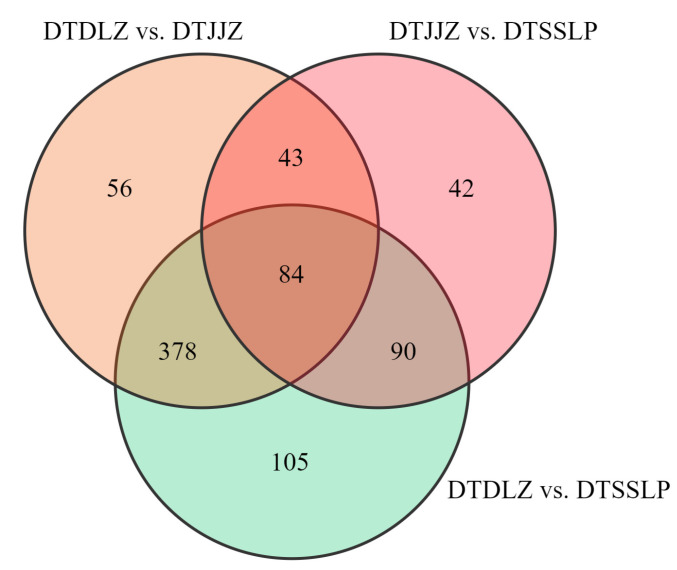
Venn diagram of differential metabolites among DTDLZ vs. DTSSLP, DTJJZ vs. DTSSLP, and DTDLZ vs. DTJJZ comparison groups.

According to the conditions of FC > 2 or <0.5 and VIP > 1, there were 657 differential metabolites identified between DTSSLP and DTDLZ, with 206 upregulated and 451 downregulated compounds (Figure 3a, Table S2). A total of 259 differential metabolites were found between DTSSLP and DTJJZ, comprising 146 upregulated and 113 downregulated metabolites (Figure 3c, Table S3). Additionally, 539 differential metabolites were observed between DTSSLP and DTDLZ, with 171 upregulated and 368 downregulated metabolites (Figure 3e, Table S4). Further categorization of the differential metabolites in different comparison groups highlighted that the main differential metabolites were amino acids and their derivatives, lipid compounds, and flavonoids between DTSSLP and DTDLZ, accounting for 35.31%, 13.55%, and 10.35%, respectively (Figure 3b). In the comparison between DTSSLP and DTJJZ, the prominent differential metabolites were amino acids and their derivatives, flavonoids, and phenolic acids, contributing to 31.27%, 16.99%, and 11.2% of the differences, respectively (Figure 3d). Similarly, significant differential metabolites included amino acids and their derivatives, lipid compounds, and flavonoids between DTDLZ and DTJJZ, representing 39.89%, 14.66%, and 10.58% of the differences, respectively (Figure 3f). Moreover, in Figure 4, a Venn diagram depicted that there were 105 unique metabolites in the DTSSLP and DTJJZ metabolite sets, 42 in the DTSSLP and DTDLZ sets, and 56 in the DTDLZ and DTJJZ sets. Additionally, there were 84 metabolites common to all three comparison groups.

### 3.5. K-Means Analysis

**Figure 5:**
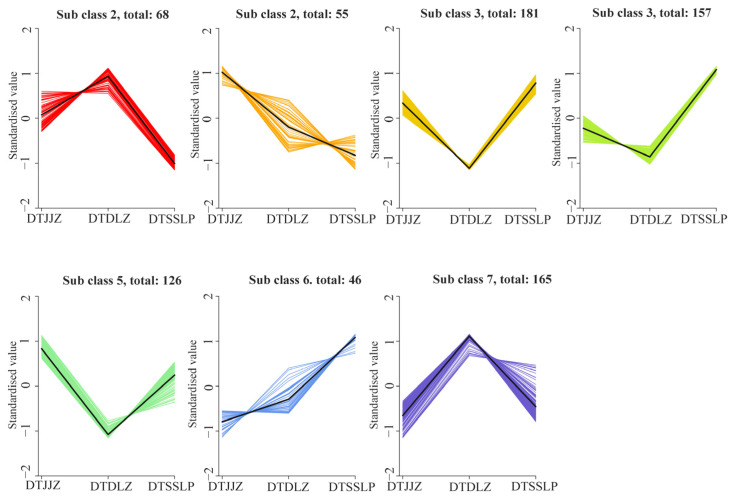
K-means plot of differential metabolites in different production areas of *H. citrina*.

In order to evaluate the difference in the relative intensity of differential metabolites among different regions of *H. citrina*, all differential metabolites were subjected to unit variance scaling. A total of 899 differential metabolites were categorized into seven subclasses using the K-means clustering method. As shown in Figure 5, the relative intensity trends of metabolites in class 1 and class 7 were roughly similar, with the highest content observed in DTDLZ. However, the metabolite content of class 1 in DTSSLP was the lowest, while that of class 7 in DTJJZ was the lowest. Notably, class 1 and class 7 contained 68 and 165 differential metabolites, respectively, with the highest proportions being amino acids and derivatives, and flavonoids. Classes 3, 4, and 5 exhibited similar trends in relative intensity, with the lowest content found in DTDLZ. However, the contents of metabolites in class 3 and 4 metabolites were the highest content in DTSSLP, whereas that of class 5 metabolites were the highest in DTJJZ. Classes 3, 4, and 5 contained 181, 157, and 126 metabolites, respectively, with the highest proportions being amino acids and derivatives with lipids, amino acids and derivatives with flavonoids, and amino acids and derivatives with nucleotides and their derivatives. Additionally, the differential metabolites present in class 2 had the highest relative intensity in DTJJZ and the lowest in DTSSLP, while class 6 showed the opposite trend. Class 2 comprised 55 metabolites, and class 6 included 46 differential metabolites, with the highest proportions being amino acids and derivatives, and phenolic acids. Overall, among 798 differential metabolites, 384 were highest in DTSSLP, 233 were highest in DTDLZ, and only 181 were highest in DTJJZ.

### 3.6. Differences in the Nutritional Components Among the Different Producing Areas of H. citrina

**Figure 6:**
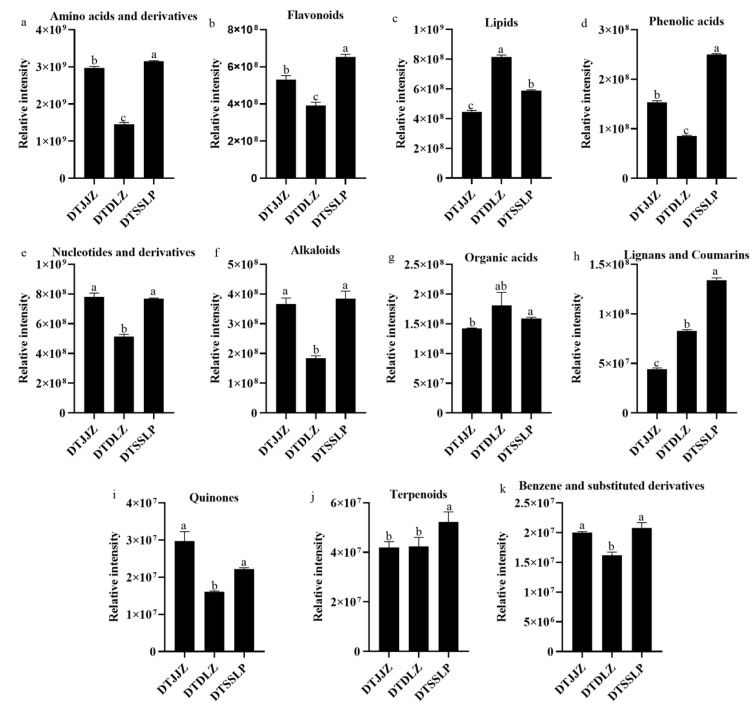
Relative intensity of metabolites with different classifications in different producing areas of *H. citrina*. (**a**): amino acids and derivatives; (**b**): flavonoids; (**c**): lipids; (**d**): phenolic acids; (**e**): nucleotides and derivatives; (**f**): alkaloids; (**g**): organic acids; (**h**): lignans and coumarins; (**i**): quinones; (**j**): terpenoids; (**k**): benzene substituted derivatives. Different letters indicate a significant difference at a 95% confidence level.

The above 798 differential metabolites comprised 272 amino acids and their derivatives, 102 flavonoids, 101 lipids, 71 phenolic acids, 57 nucleotides and their derivatives, 49 alkaloids, 35 organic acids, 28 lignans and coumarins, 17 quinones, 15 terpenoids, six benzene and substituted derivatives, and 45 others (Table S5). The characteristics and differences in the chemical composition are shown in Figure 6.

For amino acids and derivatives, DTSSLP exhibited the highest abundance, followed by DTJJZ and DTDLZ (DTSSLP > DTJJZ > DTDLZ) (Figure 6a). Specifically, 114 amino acids and their derivatives displayed higher contents in DTSSLP compared to DTJJZ and DTDLZ. Among them, the top three metabolites with the highest relative intensity in DTSSLP were Pro-Tyr-Lys, Ala-Phe-Arg, and Leu-Cys-Arg, whereas in DTDDLZ, they were Pro-Trp-Trp, Tyrosyl-cryptiophan, and Met-Asp-Arg. In DTJJZ, the top three differential metabolites in terms of relative intensity were Phe-Ala-Arg, Ala-Phe-Arg, and Pro-Tyr-Lys. Moreover, several essential amino acids exhibited higher levels in DTSSLP than in the other two regions of *H. citrina*, including L-cysteine, L-methionine, L-histidine, L-tyrosine, DL-leucine, L-lysine, L-tryptophan, and others. Previous studies have highlighted the active roles of amino acids in plant growth and their involvement in its response to environmental stresses [23]. For example, lysine catabolism plays a key role in the response to various stress factors of plants [27]. Therefore, *H. citrina* from DTSSLP may possess superior resistance to environmental stresses.

Flavonoids in DTSSLP exhibited higher abundance compared to those in DTJJZ and DTDLZ (DTSSLP > DTJJZ > DTDLZ) (Figure 6b). Specifically, 49 flavonoids demonstrated higher concentrations in DTSSLP than in DTJJZ and DTDLZ, among which quercetin, kaempferol, and their derivatives were notably prominent. Quercetin, a highly potent flavonoid renowned for its robust antioxidant properties, is commonly found in glycosylated form in various fruits and vegetables [28]. Studies indicate that acylated flavonoid glycosides derived from natural plants also have many biological activities, including antioxidant and antibacterial effects, highlighting the value of acylation in enhancing the biological properties of flavonoid glycosides [29,30]. Acylated flavonols, such as quercetin, hold promise for further development and utilization in various applications. In addition, kaempferol is present in numerous plants and foods and has garnered interest for its potential biological properties, particularly its antioxidant characteristics [31]. Therefore, *H. citrina* in DTSSLP may have better physiological efficiency than the other two regions, like antioxidant properties.

Lipids exhibited the highest abundance in DTDLZ compared to DTSSLP and DTJJZ (DTDLZ > DTSSLP > DTJJZ), as depicted in Figure 6c. The predominant lipid types included free fatty acids (FAAs), glycerol esters, lysophosphatidylcholine (LPC), lysophosphatidylethanolamine (LPE), phosphatidylcholine (PC), and sphingolipids. DTDLZ contained 44 lipids with significantly higher contents than those found in DTSSLP and DTJJZ. Notably, metabolites including LysoPC 18:0, LysoPC 18:0 (2n isomer), and LysoPC 18:1 (2n isomer) were identified as the top three in relative intensity among differential metabolites in DTDLZ. LPCs are recognized as crucial signaling molecules with diverse biological functions, including the regulation of cellular inflammation [32]. Furthermore, FAAs serve not only as essential energy sources but also as signaling molecules that regulate various cellular processes and physiological functions depending on their carbon chain length [33]. Therefore, *H. citrina* in DTDLZ may also offer enhanced physiological benefits, particularly anti-inflammatory effects.

DTSSLP exhibited the highest abundance of phenolic acids, followed by DTJJZ and DTDLZ (DTSSLP > DTJJZ > DTDLZ), as illustrated in Figure 6d. A total of 38 metabolites demonstrated significantly higher concentrations in DTSSLP compared to DTDLZ and JJZ, respectively. Among these metabolites, 4-hydroxybenzoic acid, 2-hydroxycinnamic acid*, and α-hydroxycinnamic acid* were the most abundant phenolic acids in DTSSLP. Specifically, 4-hydroxybenzoic acid is noted for its antibacterial activity, while cinnamic acid and its derivatives are predominantly recognized for their anti-inflammatory properties [34,35]. Furthermore, ferulic acid exhibited higher relative intensity in *H. citrina* of DTSSLP compared to DTDLZ and DLJJZ. Studies have highlighted its efficacy in managing diabetes, coronary heart disease, stroke, and cardiovascular diseases [36]. Thus, the elevated levels of phenolic acids in DTSSLP contributed significantly to its enhanced physiological benefits.

DTSSLP and DTJJZ exhibited the highest contents of nucleotides and their derivatives compared to DTDLZ (DTSSLP, DTJJZ > DTDLZ) (Figure 6e). Specifically, DTSSLP and DTJJZ contained 23 and 27 nucleotides and their derivatives in significantly higher contents than DTDLZ, respectively. As for alkaloids, they are basic nitrogen-containing compounds known for a bitter taste [37]. In this study, 49 alkaloids were identified in *H. citrina*, categorized into seven subclasses: Alkaloids, phenolamine, plumerane, pyridine alkaloids, pyrrole alkaloids, quinoline alkaloids, and steroid alkaloids (Figure 6f). DTSSLP and DTJJZ contained more alkaloids than DTDLZ (DTSSLP > DTJJZ > DTDLZ), with 29 and 15 alkaloids found in higher concentrations in DTSSLP and DTJJZ compared to DTDLZ, respectively.

The total relative intensity of organic acids was significantly higher in DTSSLP and DTDLZ than in DTJJZ (DTDLZ, DTSSLP > DTJJZ) (Figure 6g). There was no significant difference in organic acid content between DTDLZ and DTSSLP. Specifically, DTDLZ exhibited nine organic metabolites at higher concentrations compared to DTSSLP and DTJJZ, respectively. In contrast, DTSSLP contained 20 organic metabolites in higher concentrations compared to both DTDLZ and DTJJZ. Organic acids are known to enhance the response to long-term drought stress in plants [38]. Therefore, *H. citrina* in DTSSLP and DTDLZ may demonstrate increased tolerance to drought stress. In addition, DTSSLP exhibited the highest concentrations of lignans and coumarins. Specifically, 14 lignans and coumarins were found at higher concentrations in DTSSLP compared to both DTDLZ and DTJJZ (DTSSLP > DTDLZ > DTJJZ) (Figure 6h).

Quinones were found in the highest abundance in DTJJZ and DTSSLP compared to DTDLZ (DTJJZ, DTSSLP > DTDLZ). Specifically, DTSSLP and DTJJZ contained eight and six metabolites, respectively, at significantly higher concentrations than DTDLZ (Figure 6i). For terpenoids, DTSSLP exhibited a higher concentration compared to DTDLZ and DTJJZ. In DTSSLP, nine terpenoids were found in significantly higher concentrations than in both DTDLZ and DTJJZ, respectively (Figure 6j). While benzene and substituted derivatives generally showed higher concentrations in DTDLZ compared to DTSSLP and DTJJZ, the total relative content of these compounds was significantly greater in DTSSLP and DTJJZ compared to DTDLZ (Figure 6k).

Overall, DTSSLP showed higher concentrations of amino acids and derivatives, flavonoids, phenolic acids, lignans and coumarins, and terpenoids compared to DTDLZ and DTJJZ. However, alkaloids, nucleotides and their derivatives, benzene and substituted derivatives, and quinones exhibited higher concentrations in DTSSLP and DTJJZ compared to DTDLZ. None of the mentioned metabolites showed significant differences between DTSSLP and DTJJZ. Additionally, DTDLZ generally exhibited the lowest concentrations of these compounds, including amino acids and derivatives, flavonoids, phenolic acids, terpenoids, alkaloids, nucleotides and their derivatives, benzene and substituted derivatives, and quinones. Notably, lipids and organic acids showed higher intensities in DTDLZ compared to DTSSLP and DTJJZ. These variations in chemical profiles could be attributed to environmental factors and planting patterns.

### 3.7. KEGG Enrichment Analysis

**Figure 7:**
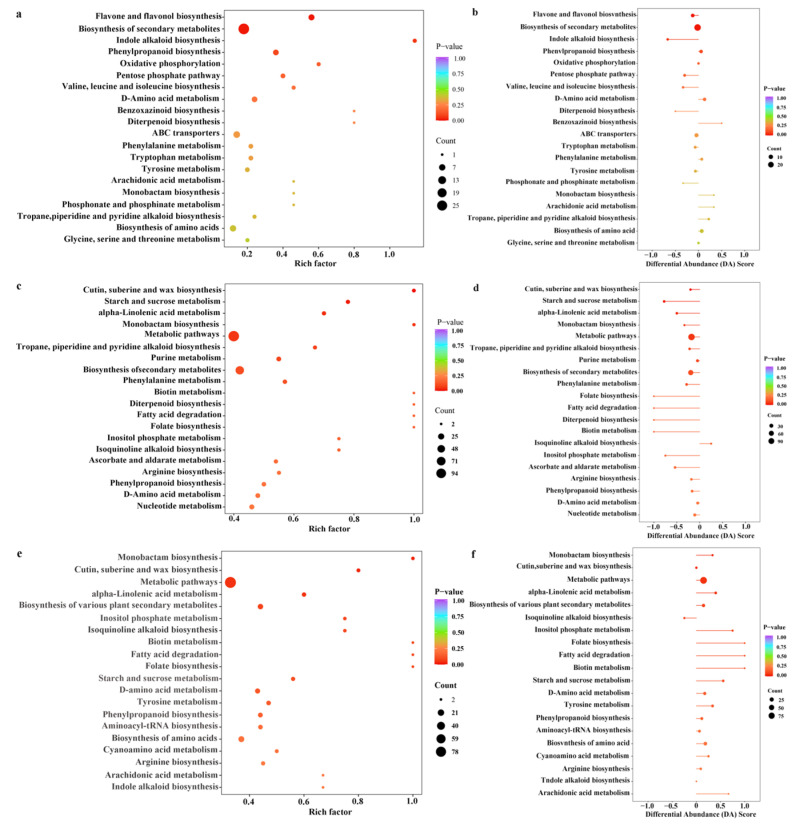
(**a**,**c**,**e**) KEGG pathway enrichment of differential metabolites between different comparison groups (DTDLZ vs. DTSSLP, DTJJZ vs. DTSSLP, DTDLZ vs. DTJJZ); (**b**,**d**,**f**) differential abundance (DA) score of differential metabolic pathways among different comparison groups (DTDLZ vs. DTSSLP, DTJJZ vs. DTSSLP, and DTDLZ vs. DTJJZ).

We conducted an enrichment analysis of differential metabolites across three comparison groups. Specifically, significant differences were observed in the metabolic pathways enriched with these metabolites between the DTSSLP and DTJJZ groups. Notably, the pathways that showed significant enrichment included flavonoid and flavonol biosynthesis, as well as the biosynthesis of secondary metabolites (Figure 7a, Table 3). Moreover, the metabolic pathways significantly enriched between DTSSLP and DTDLZ comprised cutin, suberine, and wax biosynthesis, starch and sucrose metabolism, and alpha-linolenic acid metabolism (Figure 7c, Table 3). Additionally, the differential metabolites between DTDLZ and DTJJZ exhibited significant enrichment in monobactam biosynthesis, cutin, suberine, and wax biosynthesis, metabolic pathways, and alpha-linolenic acid metabolism (Figure 7e, Table 3). To demonstrate overall trends in metabolic pathway changes, we utilized differential abundance (DA) scores. These scores indicate whether a metabolic pathway is upregulated (DA score of 1) or downregulated (DA score of −1). The length of the line in the figures represents the magnitude of the DA score, and the size of the points at the line ends corresponds to the number of differentially expressed metabolites within that pathway. Points positioned to the left or right of the axis indicate downregulation or upregulation of pathway expression, respectively. Longer lines suggest a more pronounced trend. The color of the lines and points reflects the significance level (*p*-value) of these changes, with red indicating smaller *p*-values and purple indicating larger *p*-values [39]. The DA scores illustrated that compared to DTSSLP, both DTJJZ and DTDLZ exhibited downregulation in differential metabolic pathways. Interestingly, enriched metabolic pathways showed upregulation in DTDLZ compared to DTJJZ (Figure 7b,d,f), indicating distinct metabolic responses between these two groups. Overall, these findings highlight the nuanced differences in metabolic pathway enrichment and regulation across the studied comparison groups, providing insights into the metabolic dynamics underpinning the observed biological responses.

### 3.8. Analysis of Enriched Metabolites in KEGG Pathway

**Figure 8:**
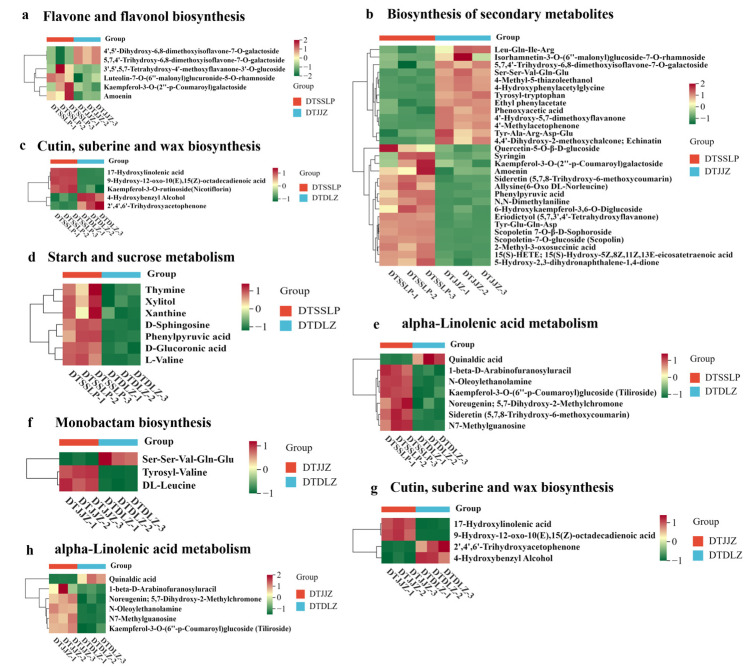
Heat map of enriched metabolites in the key KEGG pathway ((**a**,**b**) DTDLZ vs. DTSSLP; (**c**–**e**) DTJJZ vs. DTSSLP; (**f**–**h**) DTDLZ vs. DTJJZ).

The comparison of differential metabolites enriched in various metabolic pathways between different groups are illustrated in Figure 8. Specifically, six differential metabolites were identified to be enriched in flavonoid and flavonol biosynthesis between DTSSLP and DTJJZ. In DTJJZ, the levels of 3′,5′,5,7-tetrahydroxy-4′-methoxyflavanone-3′-O-glucoside, luteolin-7-O-(6″-malonyl) glucuronide-5-O-rhamnoside, and kaempferol-3-O-(2″-p-coumaroyl) galactoside* were observed to be lower compared to DTSSLP. Conversely, the levels of 4′,5′-dihydroxy-6,8-dimethoxyisoflavone-7-O-galactoside* and 5,7,4′-trihydroxy-6,8-dimethoxyisoflavone-7-O-galactoside* were higher in DTJJZ than in DTSSLP. Among the 29 differential metabolites enriched in the biosynthesis of secondary metabolites between DTSSLP and DTJJZ, 11 metabolites, such as 4′-hydroxy-5,7-dimethoxyflavanone and Tyr-Ala-Arg-Asp-Glu, were found to be at lower levels in DTJJZ compared to DTSSLP. Meanwhile, 18 metabolites, including 4′,5′-dihydroxy-6,8-dimethoxyisoflavone-7-O-galactoside* and 5,7,4′-trihydroxy-6,8-dimethoxyisoflavone-7-O-galactoside*, exhibited higher levels in DTJJZ than in DTSSLP. Flavonoid compounds in *H. citrina* are recognized for their beneficial effects on depression, sleep improvement, and lactation promotion, as documented in recent studies [6,8]. Moreover, plant secondary metabolites play pivotal roles in enhancing plant growth and establishing ecological interactions with other species, aiding plants in defense against pathogens and environmental stressors [40]. Consequently, *H. citrina* in DTSSLP potentially offered superior health benefits and resilience to environmental stresses compared to those in DTJJZ.

Examining the differential metabolites enriched in cutin, suberine, and wax biosynthesis between DTSSLP and DTDLZ, it was found that levels of 17-hydroxylinolenic acid, 9-hydroxy-12-oxo-10(E),15(Z)-octadecadienoic acid, and kaempferol-3-O-rutinoside (Nicotiflorin) were lower in DTDLZ than in DTSSLP. Conversely, levels of 4-hydroxybenzyl alcohol and 2′,4′,6′-trihydroxyphenylacetonitrile were higher in DTDLZ compared to DTSSLP. Cutin, suberin, and wax are crucial components contributing to plant cell wall synthesis, thereby enhancing the plant’s ability to withstand environmental stressors [41]. The higher levels of these metabolites in DTSSLP likely contribute to the robustness of its cell walls. In starch and sucrose metabolism, all metabolite levels were lower in DTDLZ than in DTSSLP, while six metabolites in alpha-linolenic acid metabolism (1-β-D-arabinofuranosyluracil, quercetin-3-O-(6″-p-coumaroyl) glucoside (Tiliroside), and N-Oleoylethanolamine, etc.) were found to be at lower levels in DTDLZ compared to DTSSLP. Starch and sucrose metabolism are critical for plants to respond to salt stress [42], whereas alpha-linolenic acid regulates plant immune functions. Thus, the enrichment of these metabolites in DTSSLP likely contributed to its enhanced immune response and capacity to cope with environmental stressors [43], aligning with the findings illustrated in Figure 8.

Furthermore, as indicated in Table 3, the differential metabolites between DTJJZ and DTDLZ were predominantly enriched in monobactam biosynthesis, cutin, suberine, and wax biosynthesis, metabolic pathways, and alpha-linolenic acid metabolism. The majority of differential metabolites were more abundant in DTJJZ compared to DTDLZ, suggesting that *H. citrina* in DTJJZ also exhibited superior physiological functions and characteristics to adapt to environmental stresses.

## 4. Conclusions

This study conducted TPC, TFC, and amino acid analyses to differentiate *H. citrina* from various production areas in Datong City. Additionally, a widely targeted metabolomic analysis using UPLC–ESI–QTRAP–MS/MS comprehensively assessed the nutritional composition of different *H. citrina* samples. *H. citrina* in DTSSLP had the highest contents of protein and AAs as well as TFCs and TPCs. A total of 798 differential metabolites were identified among *H. citrina* samples from distinct regions. Notably, DTSSLP exhibited higher concentrations of amino acids and derivatives, flavonoids, phenolic acids, lignans and coumarins, and terpenoids compared to DTDLZ and DTJJZ. Conversely, alkaloids, nucleotides and their derivatives, benzene and substituted derivatives, and quinones showed higher concentrations in DTSSLP and DTJJZ than in DTDLZ. DTDLZ generally displayed the lowest concentrations of compounds such as amino acids and derivatives, flavonoids, phenolic acids, terpenoids, alkaloids, nucleotides and their derivatives, benzene and substituted derivatives, and quinones. Lipids and organic acids were the exceptions, exhibiting the highest intensities in DLDLZ compared to DTSSLP and DTJJZ. In addition, according to rich factor and DA scores, pathway enrichment analysis revealed nine metabolic pathways were related with the different characteristics among DTSSLP, DTDLZ, and DTJJZ. Moreover, DA scores indicated that both DTJJZ and DTDLZ show downregulation in differential metabolic pathways compared to DTSSLP. Overall, in the present study, *H. citrina* of DTSSLP possessed the highest content of nutrients and metabolites, suggesting it may possess excellent nutritional functions, including anti-depression, sleep-promoting functions, along with better flavor and the ability to resist environmental stress. This study elucidated the composition and metabolic pathways of differential metabolites in *H. citrina* sourced from various areas of Datong City. It will serve as a valuable guideline for further research on the nutritional components and health benefits of *H. citrina*, contributing to its sustainable development. Additionally, widely targeted metabolomics provided a detailed and efficient new approach for the component analysis of *H. citrina* from different production areas. However, there are still challenges in data processing and analysis complexity, as well as standardization and result discrepancies amongst different laboratories and equipment platforms. As technology continues to advance and interdisciplinary collaboration increases, its application in distinguishing the quality and function of *H. citrina* from different regions will undoubtedly become more widespread and in-depth.

## Figures and Tables

**Table 1:** TPC, TFC, and protein content of *H. citrina* in DTSSLP, DTDLZ, and DTJJZ areas.

Group	TPC/ mg GAE·g^−1^	TFC/ mg RE·g^−1^	Protein/ g·100 g^−1^
DTSSLP	14.33 ± 0.25 a	53.95 ± 0.58 a	17.15 ± 0.27 a
DTDLZ	13.67 ± 0.25 b	52.13 ± 0.48 b	16.90 ± 0.19 ab
DTJJZ	13.85 ± 0.18 b	51.18 ± 0.35 b	16.39 ± 0.26 b

Note: Different letters indicate a significant difference at a 95% confidence level.

**Table 2:** Amino acid contents of *H. citrina* in DTSSLP, DTDLZ, and DTJJZ (unit: g/100 g *H. citrina* sample).

	DTSSLP	DTDLZ	DLJJZ
Asp	0.89 ± 0.02 a	0.54 ± 0.01 b	0.23 ± 0.01 c
Thr	0.05 ± 0.00 c	0.11 ± 0.00 a	0.09 ± 0.01 b
Ser	0.12 ± 0.00 a	0.09 ± 0.00 b	0.11 ± 0.01 ab
Glu	0.40 ± 0.01	0.39 ± 0.01	0.39 ± 0.02
Gly	0.14 ± 0.01 a	0.12 ± 0.00 b	0.11 ± 0.01 c
Ala	0.21 ± 0.02 a	0.17 ± 0.00 b	0.16 ± 0.01 b
Cys	-	-	-
Val	0.18 ± 0.00 a	0.16 ± 0.00 b	0.114 ± 0.01 c
Met	0.04 ± 0.00	0.04 ± 0.00	0.030 ± 0.00
Ile	0.15 ± 0.00 a	0.13 ± 0.00 b	0.103 ± 0.00 c
Leu	0.24 ± 0.01 a	0.19 ± 0.01 b	0.164 ± 0.01 c
Tyr	0.10 ± 0.00 a	0.09 ± 0.00 a	0.068 ± 0.01 b
Phe	0.14 ± 0.01 a	0.13 ± 0.00 a	0.09 ± 0.01 b
His	0.04 ± 0.00 a	0.04 ± 0.00 a	0.03 ± 0.00 b
Lys	0.15 ± 0.00 a	0.15 ± 0.00 ab	0.13 ± 0.02 b
Arg	0.15 ± 0.01 a	0.12 ± 0.00 b	0.11 ± 0.01 c
Pro	0.12 ± 0.00 b	0.14 ± 0.01 a	0.09 ± 0.00 c
TAAs	3.11 ± 0.06 a	2.59 ± 0.02 b	2.02 ± 0.09 c
EAAs	0.95 ± 0.02 a	0.89 ± 0.01 b	0.75 ± 0.04 c

Note: Different letters indicate a significant difference at a 95% confidence level.

**Table 3:** Differential metabolic pathway information of different comparison groups.

Groups	KEGG Pathway	ko_ID	Cluster_Frequency	*p*-Value
DTDLZ vs. DTSSLP	Flavone and flavonol biosynthesis	ko00944	6/52	0.014
	Biosynthesis of secondary metabolites	ko01110	29/52	0.015
DTDLZ vs. DTSSLP	Cutin, suberine, and wax biosynthesis	ko00073	5/141	0.007
	Starch and sucrose metabolism	ko00500	7/141	0.016
	Alpha-linolenic acid metabolism	ko00592	7/141	0.038
DTDLZ vs. DTJJZ	Monobactam biosynthesis	ko00261	3/114	0.028
	Cutin, suberine, and wax biosynthesis	ko00073	4/114	0.032
	Metabolic pathways	ko01100	95/114	0.048
	Alpha-linolenic acid metabolism	ko00592	6/114	0.049

## Data Availability

The datasets generated during the current study are included in this published article, or they are available from the corresponding authors on reasonable request.

## Supplementary Materials

The following supporting information can be downloaded at https://www.mdpi.com/article/10.3390/foods13213404/s1: Table S1: The climatic conditions of different daylily-producing areas in the year before harvesting; Table S2: Information of differential metabolites between DTDLZ and DTSSLP; Table S3: Information of differential metabolites between DTJJZ and DTSSLP; Table S4: Information of differential metabolites between DTDLZ and DTJJZ; Table S5: Metabolite information with the same pattern in different groups based on K-means analysis.
