# Supplementary material for: Widely Targeted Metabolomics Analysis Revealed the Component Differences of Hemerocallis citrina Borani in Different Production Areas of Datong
Source: Foods. 2024 Oct 25;13(21):3404. doi: 10.3390/foods13213404 (PMC11545560; doi:10.3390/foods13213404)
Supplement: Supplementary file 1 [file foods-13-03404-s001.zip › Table S1.pdf]

Supplementary material

Table S1. The climatic conditions of different daylily-producing areas in the year before harvesting

Index	Annual average temperature (℃)	Annual rainfall (mm）	Sunlight hours (h)	Altitude (m)
DTSSLP	6.4	380.6	2974	1163
DTDLZ	6.7	391.5	2987	1029
DTJJZ	7.0	399	2867	971
